# Functional expression of a proton-coupled organic cation (H^+^/OC) antiporter in human brain capillary endothelial cell line hCMEC/D3, a human blood–brain barrier model

**DOI:** 10.1186/2045-8118-10-8

**Published:** 2013-01-26

**Authors:** Keita Shimomura, Takashi Okura, Sayaka Kato, Pierre-Olivier Couraud, Jean-Michel Schermann, Tetsuya Terasaki, Yoshiharu Deguchi

**Affiliations:** 1 Department of Drug Disposition and Pharmacokinetics, School of Pharmaceutical Sciences, Teikyo University, 2-11-1 Kaga, Itabashi-ku, Tokyo, 173-8605, Japan; 2 INSERM, U1016, Institut Cochin, Paris, France; 3 CNRS, UMR8104, Paris, France; 4 Université Paris Descartes, Sorbonne Paris Cité, Paris, France; 5 Neuropsychopharmacologie des addiction (CNRS UMR 8206), Université Paris Decartes, Faculté de Pharmacie, Paris, France; 6 INSERM U705, Neuropsychopharmacologie des addiction, Paris, France; 7 Division of Membrane Transport and Drug Targeting, Graduate School of Pharmaceutical Sciences, Tohoku University, Sendai, Japan

**Keywords:** Human blood–brain barrier, Human BBB model cell, hCMEC/D3 cells, Proton-coupled organic cation antiporter, Organic cation transporter, Transport function, Diphenhydramine, Pyrilamine, Oxycodone, Active transport, Real-time PCR

## Abstract

**Background:**

Knowledge of the molecular basis and transport function of the human blood–brain barrier (BBB) is important for not only understanding human cerebral physiology, but also development of new central nervous system (CNS)-acting drugs. However, few studies have been done using human brain capillary endothelial cells, because human brain materials are difficult to obtain. The purpose of this study is to clarify the functional expression of a proton-coupled organic cation (H^+^/OC) antiporter in human brain capillary endothelial cell line hCMEC/D3, which has been recently developed as an *in vitro* human BBB model.

**Methods:**

Diphenhydramine, [^3^H]pyrilamine and oxycodone were used as cationic drugs that proved to be H^+^/OC antiporter substrates. The *in vitro* uptake experiments by hCMEC/D3 cells were carried out under several conditions.

**Results:**

Diphenhydramine and [^3^H]pyrilamine were both transported into hCMEC/D3 cells in a time- and concentration-dependent manner with K_m_ values of 59 μM and 19 μM, respectively. Each inhibited uptake of the other in a competitive manner, suggesting that a common mechanism is involved in their transport. The diphenhydramine uptake was significantly inhibited by amantadine and quinidine, but not tetraethylammonium and 1-methyl-4-phenylpyridinium (substrates for well-known organic cation transporters). The uptake was inhibited by metabolic inhibitors, but was insensitive to extracellular sodium and membrane potential. Further, the uptake was increased by extracellular alkalization and intracellular acidification. These transport properties are completely consistent with those of previously characterized H^+^/OC antiporter in rat BBB.

**Conclusions:**

The present results suggest that H^+^/OC antiporter is functionally expressed in hCMEC/D3 cells.

## Background

The human brain is protected by the existence of the blood–brain barrier (BBB), which consists of brain capillary endothelial cells linked with tight junctions [1]. It is well established that the polarized expression of numerous transporters and receptors at the brain capillary endothelial cells controls the blood–brain exchange of nutrients, waste products produced from neurotransmitter substances, and drugs [2]. Therefore, knowledge of the molecular basis and transport function of the human BBB is important for not only understanding human cerebral physiology, but also for development of new central nervous system (CNS)-acting drugs. However, few studies have been done using human brain capillary endothelial cells, because human brain materials are difficult to obtain. In addition, isolation and primary culture of brain capillary endothelial cells are laborious and time-consuming procedures [3,4]. Therefore, the development of simple *in vitro* BBB models is highly desirable.

Human immortalized brain capillary endothelial cells (hCMEC/D3) have recently been developed as an *in vitro* human BBB model [5]. This cell line has been now extensively validated by numerous laboratories worldwide in pharmacological, toxicological, immunological and infection studies. These hCMEC/D3 cells retain many of the morphological and functional characteristics of the human BBB in terms of expression of multiple transporters, receptors, tight junction proteins and various ABC transporters, including ABCB1 (MDR1/P-gp), ABCC1 (MRP1), ABCC4 (MRP4), ABCC5 (MRP5), and ABCG2 (BCRP) [2,6,7]. Furthermore, several solute carrier (SLC) transporters responsible for the blood–brain exchange of mainly nutrients, including SLC2A1 (GLUT1), SLC16A1 (MCT1), SLC29A1 (ENT1) and so on, are highly expressed at the mRNA level in this cell line [8]. On the other hand, little is known concerning the expression and function of influx transporters that may regulate the brain distribution of drugs, except for relatively abundant expression of SLCO2A1 (OATP2A1) at the mRNA level [8].

Recently, we have reported that a H^+^/OC antiporter is functionally expressed in the *in vivo* rat BBB and also in a conditionally immortalized rat BBB cell line (TR-BBB13 cells) [9,10]. This H^+^/OC antiporter mediates blood–brain transport of CNS-acting cationic drugs such as pramipexole, oxycodone and diphenhydramine, in addition to pyrilamine, in rats. A brain microdialysis study revealed that this transporter actively transports oxycodone and diphenhydramine into the brain, and their unbound concentration in brain interstitial fluid (ISF) is 3- to 5-fold higher than that in blood [10,11]. There is also evidence that clonidine [12] and methylenedioxymethamphetamine (MDMA) [13] are transported by H^+^/OC antiporter in the BBB and in peripheral cell lines, respectively. Although the molecular entity of this transporter remains unknown, the known substrates are secondary or tertiary amines with positive charge at physiological pH. This suggests that many CNS drugs used in the clinical setting may be efficiently taken up into the brain via the H^+^/OC antiporter at the BBB. In addition, this putative transporter is a potential target in the development of new CNS drugs.

The purpose of this study, therefore, is to clarify the functional expression of the H^+^/OC antiporter in hCMEC/D3 cells. We also discuss whether or not the results of *in vitro* uptake study using hCMEC/D3 cells can be extrapolated to the human BBB *in vivo*, as well as the relevance of our findings to cerebral physiology and to the development and proper use of CNS-acting cationic drugs.

## Methods

### Reagents

Diphenhydramine hydrochloride was purchased from Wako Pure Chemical Industries (Osaka, Japan), [^3^H]Pyrilamine (23 – 30 Ci/mmol) and [^14^C]inulin (2.06 mCi/g) were purchased from Amersham Bioscience (Buckingshamshire, UK) and PerkinElmer Life and Analytical Sciences, Inc. (Walthan, Massachusetts, USA), respectively. Oxycodone was kindly provided by Takeda Pharmaceutical Co. Ltd. (Osaka, Japan). All other chemicals and reagents, including diphenhydramine, were commercial products of reagent grade.

### Cell culture

The hCMEC/D3 cells had been immortalized by lentiviral transduction of the catalytic subunit of human telomerase and SV40-T antigen [5]. The cells were cultivated at 37°C in EBM-2 medium (Takara Bio, Shiga, Japan) supplemented with 2.5% fetal bovine serum, 0.025% VEGF, 0.025% R3-IGF, 0.025% hEGF, 0.01% hydrocortisone, 5 μg/mL bFGF, 1% penicillin-streptomycin and 10 mM HEPES on rat collagen type I coated dishes in 95% air and 5% CO_2_.

### Animals

Adult male Wistar rats weighing about 350 g were purchased from Japan SLC (Shizuoka, Japan); they were housed, three or four per cage, in a laboratory with free access to food and water and were maintained on a 12-hr dark/12-hr light cycle in a room with controlled temperature (24 ± 2°C) and humidity (55 ± 5%). This study was conducted according to guidelines approved by the Experimental Animal Ethical Committee of Teikyo University.

### Transport studies

The hCMEC/D3 cells used for the experiments were between passage 25 and 35. The cells were seeded on rat collagen I-coated multi-well plates (Becton Dickinson) at a density of 0.2 × 10^5^ cells/cm^2^. At 3 days after seeding the cells reached confluence, the cells were washed twice with 2 mL of phosphate-buffered saline (pH 7.4) and preincubated with the transport buffer (122 mM NaCl, 3 mM KCl, 25 mM NaHCO_3_, 1.2 mM MgSO_4_, 1.4 mM CaCl_2_, 10 mM D-glucose, 10 mM HEPES, pH 7.4) for 20 min at 37°C. After preincubation, 1 mL of the transport buffer containing diphenhydramine (30 μM) or [^3^H]pyrilamine (74 kBq/μL, 90 nM) was added to initiate uptake. The cells were incubated at 37°C for a designated time, and then washed three times with 2 mL of ice-cold incubation buffer to terminate the uptake. In the case of diphenhydramine, the cells were collected in 400 μL of 0.5% KH_2_PO_4_ solution, and stored at −20°C until HPLC determination as described below. For the determination of [^3^H]pyrilamine radioactivity, the cells were solubilized with 1 M NaOH for 60 min and the radioactivity was measured using a liquid scintillation counter after the addition of scintillation cocktail Hionic Fluor (PerkinElmer Life and Analytical Sciences). Cellular protein content was determined with a BCA protein assay kit (Pierce Chemical Co., Rochford, IL, USA).

 Uptake was expressed as the cell-to-medium ratio (μL/mg protein) obtained by dividing the uptake amount by the concentration of substrate in the transport buffer. In order to estimate transporter-mediated specific uptake parameters in the kinetic analyses, the cell-to-medium ratios for saturable components of diphenhydramine (30 – 500 μM, for 15 sec) and ^3^H]pyrilamine (6 – 500 μM, for 10 sec) were determined by subtracting the cell-to-medium ratios at the concentration of 5 mM diphenhydramine and 5 mM pyrilamine, respectively. The non-saturable uptake component can be estimated from the uptake in the presence of an excess of substrate. The initial uptake rates for saturable components were determined by multiplying the cell-to-medium ratios for saturable component by the substrate concentration in the transport buffer [9,10]. The data were fitted to the following equation to estimate the kinetic parameters by means of nonlinear least-squares regression analysis with Prism software (Graphpad, San Diego, CA, USA):

$$V=\frac{V_{\;\text{max}}\times s}{K_{m}+s}$$

where V is the initial uptake rate of substrate (nmol/min/mg protein), *s* is the substrate concentration in the medium (μM), K_m_ is the Michaelis-Menten constant (μM) and V_max_ is the maximum uptake rate (nmol/min/mg protein). V_max_/K_m_ (pmol/min/mg protein/μM = μL/min/mg protein) values were calculated as the uptake clearance for the saturable transport component.

In order to examine the energy dependency of diphenhydramine uptake by hCMEC/D3 cells, the uptake was measured as described above after pretreatment with 25 μM rotenone (dissolved in the transport medium containing 0.25% ethanol) or 0.1% NaN_3_ for 20 min. In this experiment, 10 mM D-glucose in the transport medium was replaced with 10 mM 3-O-methylglucose to reduce metabolic energy. In order to examine the sodium requirement of diphenhydramine uptake by hCMEC/D3 cells, sodium ions were replaced with *N*-methylglucamine^+^. To examine the effects of reducing the membrane potential and proton gradient on diphenhydramine uptake by hCMEC/D3 cells, 10 mM valinomycin and 10 μM carbonyl cyanide *p*-trifluoromethoxyphenylhydrazone (FCCP, a protonophore) (dissolved in the transport medium containing 0.22% ethanol), respectively, were added to the transport medium. These studies were performed in parallel with controls in the presence of the corresponding ethanol concentration. Uptake was also measured at medium pH values of 6.0, 7.4 and 8.4. When the influence of intracellular pH (pHi) was examined, the uptake was measured in the presence of 30 mM NH_4_Cl to elevate pHi [9,10]. To measure the uptake at acidic pHi, extracellular NH_4_Cl was removed after the preincubation with 30 mM NH_4_Cl, because intracellular NH_3_ rapidly diffuses out of the cells, resulting in the accumulation of protons released from NH_4_^+^ during NH_3_ generation in the cells. In the inhibition study, the uptake was measured after incubation with diphenhydramine (30 μM) for 15 sec in the presence of selected inhibitors (amantadine, quinidine, TEA, serotonin, MPP^+^ and choline) at the concentration of 1 mM. Diphenhydramine uptake (30 – 500 μM, 15 sec) was measured in the absence and presence of pyrilamine (150 μM) or oxycodone (500 μM). ^3^H]pyrilamine uptake (6 – 500 μM, 10 sec) was also measured in the absence and presence of diphenhydramine (50 μM).

### *In situ* brain perfusion study

Brain perfusion was performed by the same method as reported previously [9,14]. In brief, each rat was anesthetized and the right carotid artery was catheterized with polyethylene tubing (SP-10) filled with sodium heparin (100 IU/mL). The perfusate (Krebs-Henseleit buffer, 118 mM NaCl, 4.7 mM KCl, 25 mM NaHCO_3_, 1.2 mM KH_2_PO_4_, 2.5 mM CaCl_2_, 1.2 mM MgSO_4_, 10 mM D-glucose, pH 7.4) containing diphenhydramine (10 μM) or ^3^H]pyrilamine and ^14^C]inulin (0.9 μM), a brain intravascular marker, was passed through the catheter at the rate of 4.9 mL/min with an infusion pump (Harvard Apparatus, South Natick, MA, USA). After the infusion pump is started, 5.0 sec is required to fill the external carotid artery cannula [15]. Therefore, 5.0 sec was routinely subtracted from the gross perfusion time in each experiment, to obtain the uptake time for which the perfusate was actually within the brain capillaries. At the end of uptake for 5 – 30 sec, rats were decapitated, and the right cerebral hemisphere was dissected from the perfused brain and weighed. The brain samples were stored at −20°C until determination of diphenhydramine. The brain uptake of ^3^H]pyrilamine (1.1 nM) was also determined as described above. The radioactivity was measured using a liquid scintillation counter as described above, after solubilization of the cerebral hemisphere in Soluene-350 (PerkinElmer Life and Analytical Sciences, Boston, MA, USA) at 55°C for 3 h and decolorization by addition of 0.3 mL of 30% H_2_O_2_. The value of the permeability-surface area product (PS_BBB,inf_), which represents *in vivo* BBB permeability, was calculated after correcting for remaining intravascular diphenhydramine or ^3^H]pyrilamine, estimated from the apparent brain uptake of ^14^C]inulin [9,14].

### Determination of diphenhydramine

The collected cells in 0.5% KH_2_PO_4_ solution were homogenized by sonication. For brain tissue, the cerebral hemisphere was homogenized in 5 volumes of 0.5% KH_2_PO_4_ solution. To the homogenate (300 μL) was added orphenadrine hydrochloride (50 μM, 6.3 μL) as an internal standard, as well as 60 μL of saturated K_2_CO_3_. After mixing, samples were extracted with 1.5 mL of hexane-isopropanol (98:2, v/v) by shaking on a horizontal shaker for 15 min and then centrifuged for 10 min at 800 *g*. The upper organic layer was transferred into a tube containing 300 μL of 0.5% KH_2_PO_4_. The tube was shaken for 15 min and centrifuged for 10 min at 800 *g*. The upper organic layer was aspirated off, then 60 μL of saturated K_2_CO_3_ was added to the lower aqueous phase and the extraction step and back-extraction step were repeated. The final K_2_CO_3_ solution phase was extracted again with 1.5 mL of hexane-isopropanol and the extract was dried under a nitrogen stream. The residue was reconstituted in 100 μL of mobile phase.

Diphenhydramine was determined by ultra-performance liquid chromatography (UPLC®) with a UV detector by modification of previously reported methods [16,17]. A 7.5 μL aliquot was injected into the UPLC®. The UPLC® system (Waters ACQUITY, Milford, MA, USA) consisted of a binary solvent manager, sample manager and UV detector. The analytical column used was an ACQUITY UPLC BEH C18 (2.1 mm × 50 mm, 1.7 μm particle size, Waters). The UPLC separation was carried out at a flow rate of 0.15 mL/min with a mobile phase containing 25% acetonitrile and 0.22 M phosphate buffer. UV detection was performed at 205 nm. The retention times of diphenhydramine and orphenadrine were 6.4 and 9.1 min, respectively. The detection limit for quantification of diphenhydramine was 75 pmol.

### Expression profiling of organic cation transporters by real-time PCR

The mRNA levels of typical organic cation transporters (hOCT1-3, hOCTN1-2, hMATE1-2, hPMAT) in the hCMEC/D3 cells were measured by quantitative real-time PCR analysis. Total RNA was isolated from hCMEC/D3 cells using an RNeasy mini kit (Qiagen, Valencia, CA, USA). Single-strand cDNA was prepared from 1.0 μg of total RNA by RT (Superscript III, Invitrogen, Carlsbad, CA, USA) using random primers. Quantitative real-time PCR analysis was performed using a 7500 sequence detection system (PE Applied Biosystems, Foster City, CA, USA) with 2x SYBR Green PCR Master Mix (PE Applied Biosystems) according to the manufacturer's protocols. The primers are listed in Table 1. The thermal protocol was set to 2 min at 50°C, followed by 10 min at 95°C, and then 40 cycles of 15 sec at 95°C and 1 min at 60°C. The relative expression levels of these mRNAs were calculated using the comparative Ct method for relative quantification based on the mRNA level of the housekeeping gene encoding GAPDH, according to the manufacturer's protocols. The control, lacking the RT enzyme, was assayed in parallel to monitor genomic contamination. To confirm specificity of amplification, the PCR products were subjected to melting curve analysis.

**Table 1:** Sense and antisense primers for quantitative PCR

**Target mRNA**	**Sequence(5’-3’)**	**Product size**
**(Accession number)**		**(bp)**
hOCT1/*SLC22A1*	AATGGACCACATCGCTCAAAA	
(NM_003057)	CTTCGAGGGAAAGCATCTTTAAAT	68
hOCT2/*SLC22A2*	GGACGGCTGGGTGTACGA	
(NM_003058)	GGAGTTGGCACATACCAGGTTAA	70
hOCT3/*SLC22A3*	CATGCCTTGTCACTGCGTTCT	
(NM_021977)	ATGTAGCCACTGTGGTCCTCAA	63
hOCTN1/*SLC22A4*	CCAGAGTGGGCAGCATCAT	
(NM_003059)	TAGGGCAGCATTCTGTTGTAAGC	67
hOCTN2/*SLC22A5*	CCTTCTCTTCATGCAGCTGGTA	
(NM_003060)	CCCACCATCACCAGGACTGT	66
hMATE1/*SLC44A1*	CAGTCACGCTGGCAATCG	
(NM_080546)	GGTGTCACAGGCAGAAGATAAGC	77
hMATE2/*SLC44A2*	TCTGAGAGGAACTGGGAAGCA	
(NM_152908)	AGGCCGATGATGTAATATGTGATG	69
hPMAT/*SLC29A4*	CAGCTTTCACGGATACTACATTGG	
(NM_001040661)	GCAAAGTAGATGGCGTGATAACG	67
hGAPDH	CCACATCGCTCAGACACCAT	
(NM_002046)	GCGCCCAATACGACCAAAT	66

### Statistical analysis

Statistical analysis of the data was performed by employing Student’s *t*-test and by one-way analysis of variance followed by Dunnett’s test for single and multiple comparisons, respectively. Differences were considered statistically significant at *P* < 0.05.

## Results

### Uptake kinetics of diphenhydramine and [^3^H]pyrilamine by hCMEC/D3 cells

**Figure 1:**
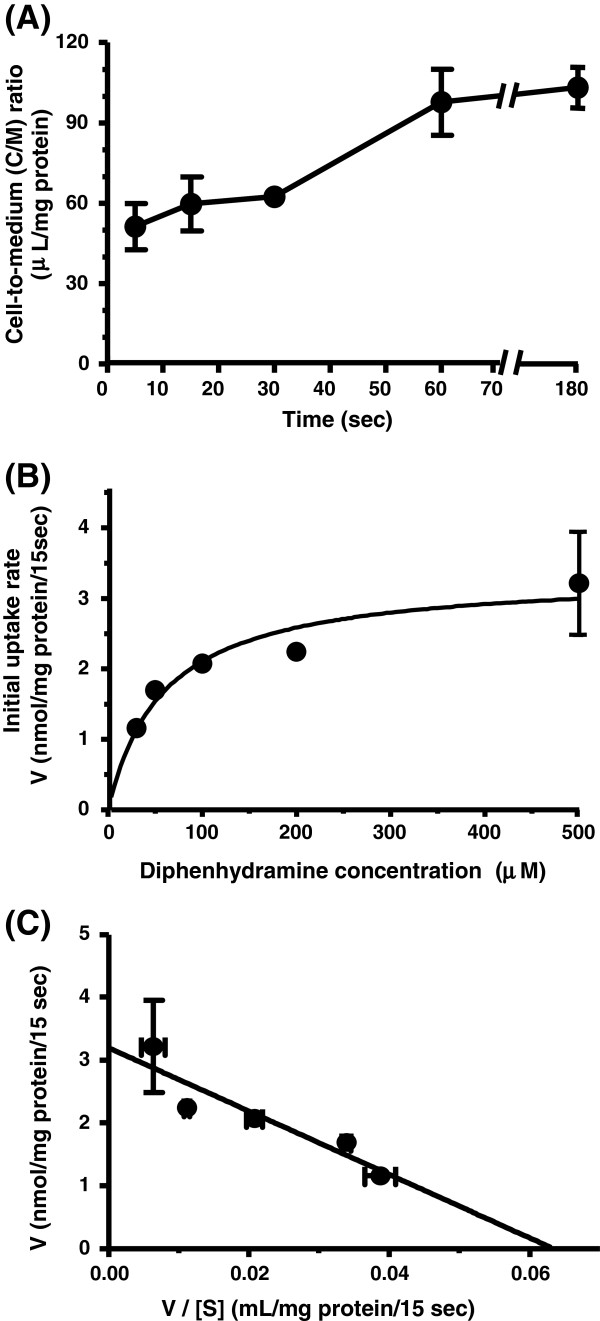
**Diphenhydramine uptake by hCMEC/D3 cells.(A)** Time course of the cell-to-medium (C/M) ratio of diphenhydramine uptake by hCMEC/D3 cells. Uptake of diphenhydramine (30 μM) was measured at 37°C. **(B)** Concentration-dependence of the initial uptake rate of diphenhydramine in hCMEC/D3 cells. hCMEC/D3 cells were preincubated with incubation buffer (122 mM NaCl, 3 mM KCl, 25 mM NaHCO_3_, 1.2 mM MgSO_4_, 1.4 mM CaCl_2_, 10 mM D-glucose, 10 mM HEPES, pH 7.4) for 20 min at 37°C. Uptake of diphenhydramine (30 μM) was measured at 37°C for 15 sec. **(C)** Eadie-Hofstee plot of diphenhydramine uptake by hCMEC/D3 cells. V, initial uptake rate (nmol/mg protein/15 sec). [S], diphenhydramine concentration in the transport medium (μM). Each point represents the mean ± S.E. from three to four determinations. When verticals are not shown, the S.E. is contained within the limits of the symbols.

**Figure 2:**
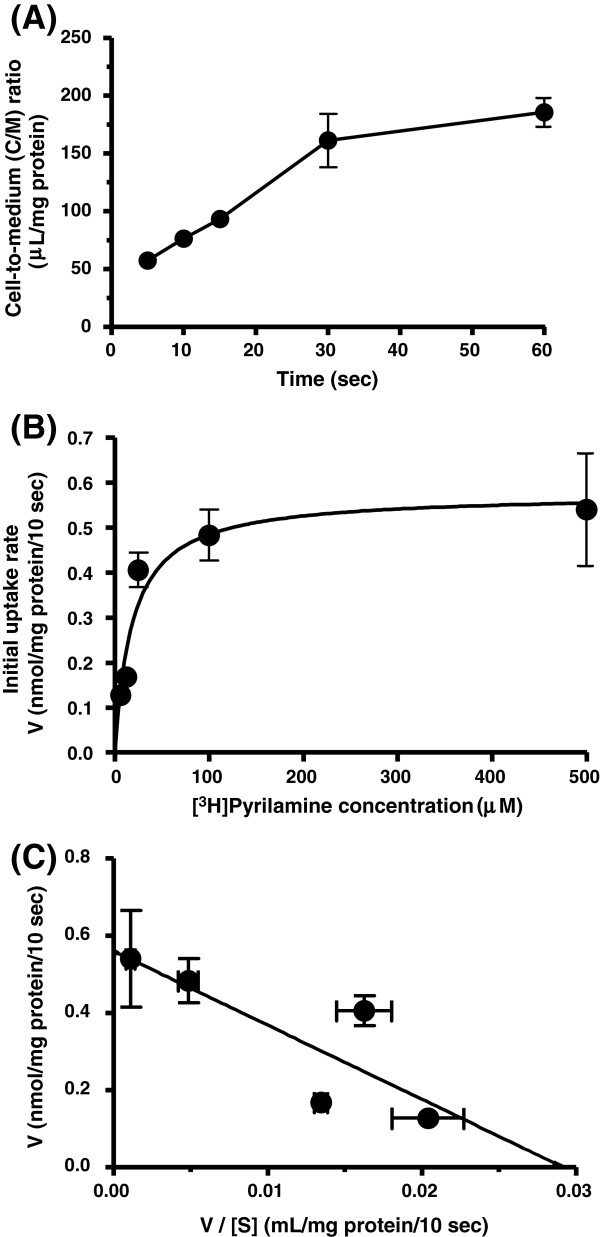
**[**^**3**^**H]pyrilamine uptake by hCMEC/D3 cells.(A)** Time course of the cell-to-medium (C/M) ratio of [^3^H]pyrilamine uptake by hCMEC/D3 cells. Uptake of [^3^H]pyrilamine (74 kBq/μL, 90 nM) was measured at 37°C. **(B)** Concentration-dependence of the initial uptake rate of [^3^H]pyrilamine in hCMEC/D3 cells. hCMEC/D3 cells were preincubated with incubation buffer (122 mM NaCl, 3 mM KCl, 25 mM NaHCO_3_, 1.2 mM MgSO_4_, 1.4 mM CaCl_2_, 10 mM D-glucose, 10 mM HEPES, pH 7.4) for 20 min at 37°C. Uptake of [^3^H]pyrilamine (74 kBq/μL, 90 nM) was measured at 37°C for 10 sec. **(C)** Eadie-Hofstee plot of [^3^H]pyrilamine uptake by hCMEC/D3 cells. V, initial uptake rate (nmol/mg protein/10 sec). [S], [^3^H]pyrilamine concentration in the transport medium (μM). Each point represents the mean ± S.E. from three to four determinations. When verticals are not shown, the S.E. is contained within the limits of the symbols.

The uptake of diphenhydramine (30 μM) increased in proportion with time until 60 sec at 37°C, and reached equilibrium with the cell-to-medium (C/M) ratio of 97.7 – 103 μL/mg protein at 60 – 180 sec (Figure 1A). ^3^H]Pyrilamine uptake (74 kBq/μL, 90 nM) by hCMEC/D3 cells also increased linearly with time until 30 sec, and the C/M ratio was 161 – 186 μL/mg protein at 30 – 60 sec (Figure 2A). Therefore, the initial uptake rate was assessed at 15 sec for diphenhydramine and 10 sec for ^3^H]pyrilamine, in the subsequent kinetic and inhibition studies. The initial uptakes of diphenhydramine and ^3^H]pyrilamine were concentration-dependent (Figures 1B and 2B). Eadie-Hofstee plot for diphenhydramine uptake gave a single straight line (r^2^ = 0.859), indicating involvement of a single saturable process (Figure 1C). Although the Eadie-Hofstee plot for ^3^H]pyrilamine uptake has the lower r^2^ value (0.679) than that for diphenhydramine, the kinetic parameters for both drugs were analyzed assuming that each drug has a single saturable transport process, on the basis of our previous reports [9,10]. Kinetic analysis provided a K_m_ value of 59 μM and a V_max_ of 13 nmol/mg protein/min for diphenhydramine, and a K_m_ value of 19 μM and a V_max_ of 3.5 nmol/mg protein/min for ^3^H]pyrilamine. The V_max_/K_m_ values (uptake clearance) for diphenhydramine and ^3^H]pyrilamine were 220 and 184 (μL/min/mg protein), respectively.

### Metabolic energy and ion dependence of the uptake of diphenhydramine by hCMEC/D3 cells

Diphenhydramine uptake in hCMEC/D3 cells was significantly inhibited by pretreatment with rotenone and sodium azide, but was not affected by replacement of extracellular sodium ion with *N*-methylglucamine^+^ or by treatment with valinomycin, a potassium ionophore (Table 2).

**Table 2:** Effects of metabolic inhibitors (rotenone and sodium azide), protonophore (FCCP), sodium replacement and change in membrane potential by valinomycin on diphenhydramine uptake by hCMEC/D3 cells

**Treatment**	**Relative uptake (% of control)**
Rotenone (25 μM) ^a),^^b)^	29.6 ± 26.9 **
Sodium azide (0.1%) ^b)^	11.8 ± 0.4 **
10 μM FCCP	30.6 ± 1.3 ***
Na^+^ replacement	95.3 ± 4.5
Valinomycin (10 μM) ^c)^	84.1 ± 0.2

Uptake of diphenhydramine (30 μM) was measured at 37°C for 15 sec in the absence or presence of metabolic inhibitors (25 μM rotenone and 0.1% sodium azide) and a protonophore (10 μM p- FCCP) and 10 µM valinomycin. The uptake was also measured in sodium-free incubation buffer (Na^+^ was replaced with N-methylglucamine). ^a)^ Rotenone was dissolved in the transport buffer containing 0.25% ethanol. ^b)^ 25 µM rotenone and 0.1% sodium azide were added to the transport buffer without 10 mM D-glucose to reduce metabolic energy. ^c)^ The cells were preincubated with the transport buffer containing 10 μM valinomycin for 10 min. Valinomycin was dissolved in the transport buffer containing 0.22% ethanol. Each study was performed in parallel with a control containing the corresponding ethanol concentration. Each value represents the mean as % of control ± S.E. (n = 3). **p<0.01 and **p<0.001 *vs* control.

**Figure 3:**
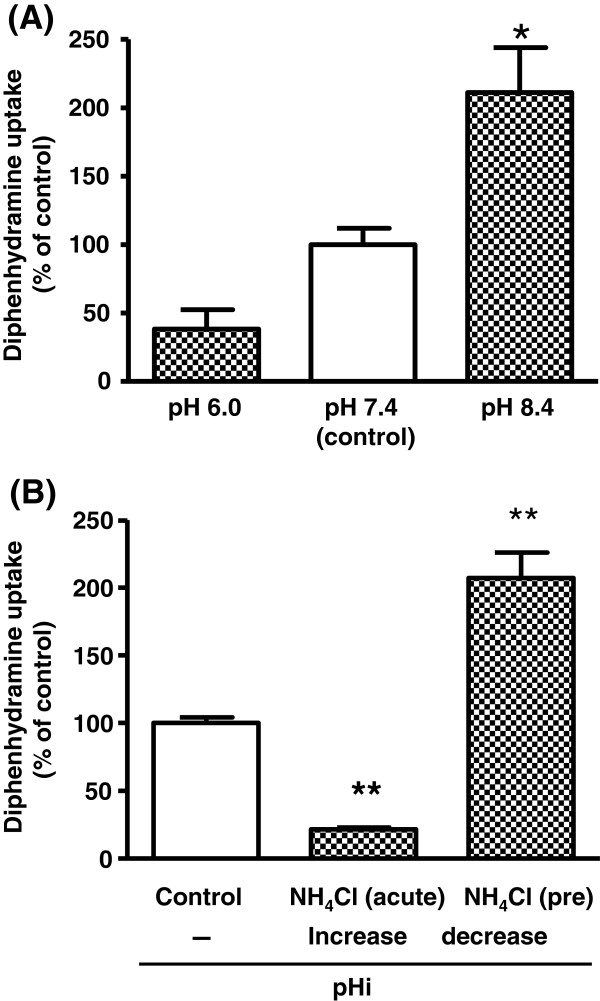
**Effect of extracellular pH and intracellular pH on diphenhydramine uptake by hCMEC/D3 cells.(A)** Uptake of diphenhydramine (30 μM) was measured at 37°C for 15 sec at medium pH values of 6.0, 7.4 and 8.4. Each column represents the mean ± S.E. of three determinations. Asterisks show a significant difference, *P<0.05 *vs* pH 7.4 (control). **(B)** Uptake of diphenhydramine (30 μM) was measured at 37°C, pH 7.4 for 15 sec. The uptake was measured in the presence of 30 mM NH_4_Cl to elevate intracellular pH (pHi) (NH_4_Cl (acute)). To measure the uptake at acidic pHi, extracellular NH_4_Cl was removed after preincubation with 30 mM NH_4_Cl for 20 min (NH_4_Cl (pre)). Each column represents the mean ± S.E. of three determinations. Asterisks show a significant difference, **P<0.01 *vs* control.

The C/M ratio of diphenhydramine was decreased in acidic transport medium (pH 6.0) and significantly increased in alkaline medium (pH 8.4) by 2.1-fold, compared with that at pH 7.4 (Figure 3A); it was also decreased by treatment with FCCP, a protonophore (Table 2). To examine the effect of protons as a driving force, the cells were treated with NH_4_Cl, because the intracellular pH (pHi) value rises in the presence of NH_4_Cl (acute treatment), whereas pretreatment and subsequent removal of NH_4_Cl (pretreatment) causes a decrease in the pHi value. Intracellular alkalization markedly reduced diphenhydramine uptake, while intracellular acidification resulted in stimulation of diphenhydramine uptake (Figure 3B).

### Inhibition of uptake of diphenhydramine and [^3^H]pyrilamine by hCMEC/D3 cells

**Figure 4:**
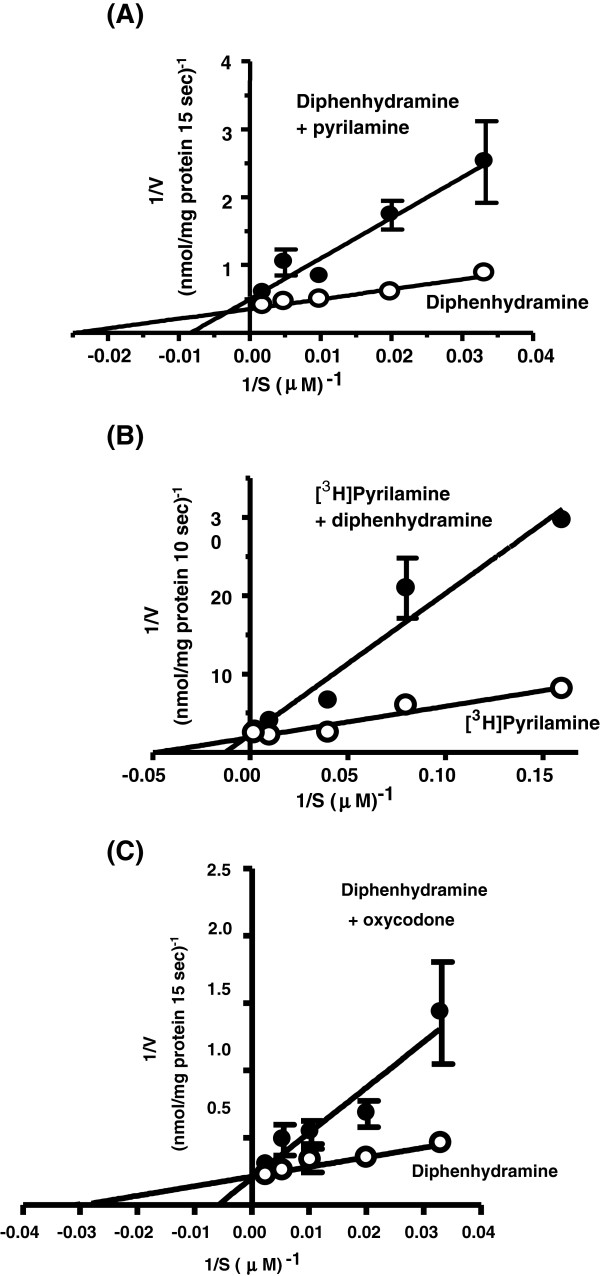
**Lineweaver-Burk plot of mutual inhibitory effects on uptakes of diphenhydramine or [**^**3**^**H]pyrilamine by hCMEC/D3 cells.** Diphenhydramine uptake was measured at 37°C for 15 sec in the presence (●) or absence (○) of pyrilamine (150 μM) **(A)** or oxycodone (500 μM) **(C)**. [^3^H]Pyrilamine uptake was also measured at 37°C for 10 sec in the presence (●) or absence (○) of diphenhydramine (50 μM) **(B)**. Each point represents the mean ± S.E. of four determinations.

In Lineweaver-Burk plot analyses of mutual inhibitory effects on uptake of diphenhydramine and pyrilamine (Figure 4), the plots of diphenhydramine uptake in the presence and absence of pyrilamine intersected at the ordinate axis (Figure 4A). This result indicated that pyrilamine competitively inhibited diphenhydramine uptake with a K_i_ value of 240 μM. Diphenhydramine also competitively inhibited [^3^H]pyrilamine uptake with a K_i_ value of 28.9 μM (Figure 4B). These mutual and competitive inhibitions between diphenhydramine and pyrilamine suggest that a common transporter is involved in their influx transport into the hCMEC/D3 cells, at least in part. Further, diphenhydramine uptake was competitively inhibited by oxycodone with a K_i_ value of 304 μM (Figure 4C). In the inhibition study, diphenhydramine uptake was significantly inhibited by the cationic compounds amantadine and quinidine to less than 25% of the control, while TEA (a classical substrate and/or inhibitor of OCTs), MPP^+^ (a classical substrate and/or inhibitor of PMAT as well as OCTs), serotonin, and choline (a substrate or inhibitor of the choline transport system) had little effect (Table 3).

**Table 3:** Inhibitory effects of various compounds on diphenhydramine uptake by hCMEC/D3 cells

**Inhibitor**	**Concentration (mM)**	**Relative uptake (% of control)**		
Amantadine	1	23.7 ± 3.8 ***		
Quinidine	1	24.0 ± 6.2 ***		
TEA	1	98.9 ± 12.3		
Serotonin	1	84.7 ± 14.9		
MPP^+^	1	90.2 ± 11.4		
Choline	1	74.1 ± 7.3		

The inhibitory effects of selected compounds on diphenhydramine uptake by hCMEC/D3 cells. Uptake of diphenhydramine (30 μM) was measured at 37°C for 15 sec in the transport medium (pH 7.4) containing each compound (1 mM). Each column represents mean ± S.E. of three determinations. Asterisks show a significant difference, *** P < 0.01 *vs* control.

### mRNA expression of organic cation transporters in hCMEC/D3 cells

To provide molecular evidence for the expression of organic cation transporters in hCMEC/D3 cells, the mRNA expression levels of hOCT1, hOCT2, hOCT3, hOCTN1, hOCTN2, hMATE1, hMATE2 and hPMAT in hCMEC/D3 cells were determined by quantitative RT-PCR analysis (Table 4). Relatively high mRNA expression of hOCTN2 was found, followed by hOCTN1 and hPMAT. In contrast, little expression of hOCTs and hMATEs mRNAs was seen in hCMEC/D3 cells.

**Table 4:** mRNA expression levels of organic cation transporters in hCMEC/D3cells determined by quantitative RT-PCR analysis

**Target mRNA**	**mRNA expression**
	**(target mRNA x 10**^**5**^**/GAPDH mRNA)**
hOCT1/*SLC22A1*	1.99 ± 0.21
hOCT2/*SLC22A2*	N. D.
hOCT3/*SLC22A3*	8.59 ± 0.55
hOCTN1/*SLC22A4*	80.7 ± 4.6
hOCTN2/*SLC22A5*	248 ± 7.3
hMATE1/*SLC44A1*	0.29 ± 0.02
hMATE2/*SLC44A2*	1.15 ± 0.16
hPMAT/*SLC29A4*	34.0 ± 2.4

Each value represents the mean ± S.E. of eight determinations. N.D.: not detected.

### *In vivo* blood-to-brain transport of diphenhydramine and [^3^H]pyrilamine in rats

The brain uptakes of diphenhydramine and ^3^H]pyrilamine were measured by the *in situ* brain perfusion technique. The brain/perfusate (B/P) ratios of diphenhydramine and ^3^H]pyrilamine linearly increased with increasing perfusion time up to 30 sec. The PS_BBB,inf_ values for diphenhydramine and ^3^H]pyrilamine were 4.40 and 1.30 mL/min/g brain, respectively. The PS_BBB,inf_ value for ^3^H]pyrilamine was similar to the value reported previously (1.6 mL/min/g brain) [18].

## Discussion

There is increasing evidence that the immortalized human brain capillary endothelial cell line hCMEC/D3 is a good model to predict BBB permeability in the human brain [5,19]. In the present study, we further investigated the utility of this model by examining whether H^+^/OC antiporter is functionally expressed in the cells. Although this putative H^+^/OC antiporter still remains to be identified at the molecular level, it is known to transport several CNS-acting drugs with secondary or tertiary amine moieties, including diphenhydramine, oxycodone, pyrilamine and clonidine [9,10,12].

Diphenhydramine, pyrilamine and oxycodone each form a cation at physiological pH because they are weak bases having a tertiary amine moiety. We previously showed that diphenhydramine, pyrilamine and oxycodone are taken up via a pH-sensitive, energy-dependent, proton-coupled antiport system in TR-BBB13 cells, which are an *in vitro* rat BBB model [9,10]. In addition, both diphenhydramine and oxycodone have been reported to be actively taken up by the brain across the BBB with a Kp,uu (unbound concentration ratio of brain interstitial fluid to plasma) value of more than 3 in rats [10,11]. Consequently, we have suggested that H^+^/OC antiporter works in the rat BBB as an active influx transporter. In the present study, diphenhydramine and pyrilamine were used as substrates to investigate this activity.

The uptakes of diphenhydramine and ^3^H]pyrilamine were time- and concentration-dependent. Kinetic analyses revealed that the calculated uptake clearances (V_max_/K_m_) for diphenhydramine (220 μL/min/mg protein) and ^3^H]pyrilamine (184 μL/min/mg protein) were in good agreement with those of diphenhydramine (440 μL/min/mg protein) and ^3^H]pyrilamine (140 μL/min/mg protein) in conditionally immortalized rat brain capillary endothelial cells (TR-BBB13) [9,10]. These results suggest that a transporter with similar transport activity and transport clearance to those observed in the case of TR-BBB13 cells is involved in uptake of diphenhydramine and ^3^H]pyrilamine by hCMEC/D3 cells. Furthermore, the transporter seems to show no marked species difference between TR-BBB13 cells and hCMEC/D3 cells.

The uptake of diphenhydramine was significantly inhibited by pretreatment with metabolic inhibitors, but was insensitive to extracellular sodium and membrane potential in hCMEC/D3 cells (Table 2), suggesting the involvement of a transporter having similar energy and membrane potential dependencies to those of TR-BBB13 cells. Furthermore, the diphenhydramine uptake by hCMEC/D3 cells showed pH-dependency characteristic of a proton-coupled antiporter. The uptake was increased at higher extracellular pH (pH 8.4), and decreased in the presence of FCCP. Intracellular acidification induced with NH_4_Cl, stimulated the uptake (Figure 3 and Table 2). As the p*K*a value of diphenhydramine is 8.98, the proportion of uncharged diphenhydramine can be estimated to be 20.8% at pH 8.4, 2.6% at pH 7.4 and 0.1% at pH 6.0. Compared to the large change of the uncharged fraction (one twenty-sixth at pH 6.0 and eightfold at pH 8.4 compared with pH 7.4), the acidification (pH 6.0) or alkalization (pH 8.4) caused a small change in diphenhydramine uptake (two-fifths at pH6.0 and twofold at pH 8.4 of control uptake at pH7.4), suggesting that passive diffusion according to the pH-partition theory could not be solely responsible for diphenhydramine uptake by hCMEC/D3 cells. This view is further supported by the result that an outward proton gradient from intracellular fluid to extracellular medium effectively enhanced diphenhydramine uptake by hCMEC/D3 cells.

The results of the inhibition study (Figure 4 and Table 3) also indicate that H^+^/OC antiporter is functionally expressed in hCMEC/D3 cells. Diphenhydramine and pyrilamine each mutually inhibited the uptake of the other, suggesting the occurrence of competition between oxycodone and pyrilamine for a common transporter. Oxycodone also competitively inhibited diphenhydramine transport in hCMEC/D3 cells (Figure 4). A variety of organic cations with widely differing molecular structures (type II cations), such as pyrilamine, oxycodone, quinidine and amantadine, markedly inhibited diphenhydramine uptake by hCMEC/D3 cells (Figure 4 and Table 3). These results are consistent with those obtained in TR-BBB13 cells [9,10,20]. In contrast, TEA and serotonin, which are prototypical substrates/inhibitors of OCT1-3 and PMAT, respectively, had no significant effect. Given that the transport activities of OCT1-3 and PMAT are reduced by membrane depolarization, it is unlikely that these cation transporters are the molecular entity of the H^+^/OC antiporter. Low or negligible expression of hOCT1-2 mRNA in hCMEC/D3 cells also supports this idea (Table 4).

Quantitative RT-PCR analysis showed that the expression level of hOCTN2 was the highest in hCMEC/D3 cells, followed by hOCTN1, hPMAT, hOCT3 and hOCT1. On the other hand, the expression levels of hOCT2 and hMATE1-2 were negligible or low in hCMEC/D3 cells. Expression levels of these mRNAs in hCMEC/D3 cells were similar to those reported in TR-BBB13 cells [9] and rat brain capillary endothelial cells (RBEC1) [21]. The H^+^/OC antiporter at the BBB remains molecularly unidentified even in rodents. Because hCMEC/D3 cells possess a H^+^/OC antiporter, like rodent BBB, and show similar mRNA expression of identified organic cation transporters to those in rat BBB model cells, hCMEC/D3 cells should be a good *in vitro* model for further studies on the H^+^/OC antiporter. Kooijmans *et al.* have reported that amino acid transporter B^0,+^ (SLC6A14) is involved in Na^+^- and Cl^-^-dependent amantadine transport in hCMEC/D3 cells [22]. Although amantadine inhibited diphenhydramine transport in hCMEC/D3 cells, diphenhydramine transport was insensitive to extracellular Na^+^. Thus, an unidentified transport system different from SLC6A14 seems to be a candidate for the H^+^/OC antiporter.

An aim of this study was to investigate whether or not the results of *in vitro* uptake study using hCMEC/D3 cells can be extrapolated to the human BBB. As a first step, the influx BBB permeability-surface area product (PS_BBB,inf_) for diphenhydramine and ^3^H]pyrilamine in rats were compared with those measured by the *in vitro* uptake study using TR-BBB13 cells. The value of PS_BBB,inf_ measured by the *in situ* brain perfusion is mainly reflected in the unidirectional clearance from perfusate to brain across the BBB, as far as the BBB transport process is the rate-limiting step [15]. The values of PS_BBB,inf_ was estimated to be 44 and 13 μL/min/cm^2^ for diphenhydramine and ^3^H]pyrilamine, respectively, assuming that the rat brain capillary surface area is 100 cm^2^/g of brain [3]. These values approximate to the *in vitro* uptake clearances in TR-BBB13 cells for diphenhydramine (21 μL/min/cm^2^) [10] and ^3^H]pyrilamine (6.3 μL/min/cm^2^) [9]. These results indicate the possibility that the *in vivo* BBB permeability can be roughly predicted from the *in vitro* uptake clearance estimated by BBB model cells, as far as diphenhydramine and pyrilamine. In hCMEC/D3 cells, *in vitro* uptake clearance for ^3^H]pyrilamine is estimated to be 8.39 μL/min/cm^2^, which is in fairly good agreement with the *in vivo* human BBB of ^11^C]pyrilamine (15 μL/min/cm^2^) estimated from a positron emission tomography (PET) study [23]. Extensive further studies and human data will be needed to allow reliable prediction of human BBB permeability from *in vitro* uptake studies using hCMEC/D3 cells.

## Conclusion

Our results strongly suggest that H^+^/OC antiporter is functionally expressed in the immortalized human brain capillary endothelial cell line hCMEC/D3. Like the putative H^+^/OC antiporter in rodents, the transporter was energy-dependent and also dependent on an oppositely directed proton gradient, but was sodium ion- or membrane potential-independent. These findings should be relevant to the development and clinical application of CNS-acting cationic drugs in humans. We suggest that the hCMEC/D3 cell line should be a useful model system in the development of new CNS-acting drugs and optimal pharmacotherapy for various CNS diseases.

## Abbreviations

BBB: Blood–brain barrier; FCCP: carbonyl cyanide*p*-trifluoromethoxyphenylhydrazone; MPP^+^: 1-methyl-4-phenylpyridinium; H^+^/OC: Proton-coupled organic cation; TEA: Tetraethylammonium; PMAT: Plasma membrane monoamine transporter; CNS: Central nervous system; ISF: Brain interstitial fluid; MDMA: Methylenedioxymethamphetamine.

## Competing interests

The authors declare that they have no competing interests.

## Authors’ contributions

KS and TO carried out the *in vitro* transport studies. SK carried out the *in vivo* animal study. TO carried out the manuscript preparation. PC helped to draft the manuscript, and supplied hCMEC/D3 cells under license from INSERM. JS and TT helped to draft the manuscript. YD supervised the study design and manuscript preparation. All authors read and approved the final manuscript.
